# Asymmetric Differences in the Gray Matter Volume and Functional Connections of the Amygdala Are Associated With Clinical Manifestations of Alzheimer’s Disease

**DOI:** 10.3389/fnins.2020.00602

**Published:** 2020-06-26

**Authors:** Xingqi Wu, Yue Wu, Zhi Geng, Shanshan Zhou, Ling Wei, Gong-Jun Ji, Yanghua Tian, Kai Wang

**Affiliations:** ^1^Department of Neurology, The First Affiliated Hospital of Anhui Medical University, Hefei, China; ^2^Anhui Province Key Laboratory of Cognition and Neuropsychiatric Disorders, Hefei, China; ^3^Collaborative Innovation Center of Neuropsychiatric Disorders and Mental Health, Hefei, China; ^4^Department of Medical Psychology, The First Affiliated Hospital of Anhui Medical University, Hefei, China

**Keywords:** hemispheric asymmetry, voxel-based morphometry, resting-state functional magnetic resonance imaging, Alzheimer’s disease, amygdala, thalamus

## Abstract

**Objective:**

Asymmetry is a subtle but pervasive aspect of the human brain, which may be altered in several neuropsychiatric conditions. Magnetic resonance imaging (MRI) studies have reported that cerebral structural asymmetries are altered in Alzheimer’s disease (AD), but most of these studies were conducted at the region-of-interest level. At the functional level, there are few reports of resting-state functional asymmetries based on functional MRI. In this study, we investigated lateral differences in structural volumes and strengths of functional connectivity between individuals with AD and healthy controls (HCs) at the voxel level.

**Methods:**

Forty-eight patients with AD and 32 matched HCs were assessed. An analysis of voxel-based morphometry (VBM) of gray matter volume was performed at the whole-brain level to explore anatomical cerebral asymmetries in AD. We then performed a seed-to-whole-brain functional connectivity (FC) analysis to reveal FC asymmetries in AD. An asymmetry index (AI) was used to measure these changes, and the relationship between the structural and functional AIs and the clinical symptoms of AD was explored.

**Results:**

A VBM analysis revealed a rightward and a leftward lateralization in the amygdala and the thalamus, respectively, in patients with AD. FC between the amygdala and the precuneus showed a rightward lateralization in AD, which was the opposite of the lateralization in the HCs. The asymmetric changes in structure and function were associated with disease severity and functional impairment in AD.

**Conclusion:**

Our study highlights the value of considering asymmetries in the amygdala and the thalamus in clinical evaluations and their relevance to clinical measures.

## Introduction

Alzheimer’s disease (AD) is a neurodegenerative disorder that presents with diffuse brain atrophy (Sarica et al., 2018). Initial pathological changes reportedly occur in layer II of the entorhinal cortex and subsequently spread to the whole brain (Braak and Braak, 1991). The pathological progression of AD does not affect both brain hemispheres equally. Previous studies demonstrated that neuronal loss occurred earlier and progressed faster in the left hemisphere than in the right hemisphere in patients with AD; in other words, the left hemisphere has a higher susceptibility to neurodegeneration (Thompson et al., 2001, 2003; Wachinger et al., 2016; Minkova et al., 2018). Thus, it was speculated that lateralization in patients with AD differs from that in healthy controls (HCs); this speculation has been confirmed by postmortem neuroanatomical studies and *in vivo* neuroimaging.

Indeed a large number of morphology-based studies have reported a variety of regional abnormalities in hemispheric asymmetry in AD (Low et al., 2019). Wachinger et al. (2016) found asymmetric abnormalities in the shape of the hippocampus and the amygdala of patients with AD compared with healthy controls (HCs). Sarica and colleagues used regions of interest (ROIs) to show that asymmetries of the hippocampal subfields varied between AD patients and HCs (Sarica et al., 2018). Recently, a thalamic subregion study revealed that the overall asymmetry of the thalamus did not differ between groups, but a greater leftward lateralization of atrophy was observed in the ventral nuclei in patients with AD compared with HCs (Low et al., 2019). All previous studies showed that the characteristic cerebral atrophy observed in AD was bilateral, but the changes may not be symmetrical between hemispheres. However, most prior studies were based on ROIs; hence, many potential asymmetric alterations could have been overlooked. Therefore, further whole-brain analysis through voxel-based morphometry (VBM) is necessary.

Although the prevalence of asymmetrical cerebral alterations has been well established across the brain in AD, it is still unclear how hemispheric differences alter the functional networks associated with asymmetrically altered structures between hemispheres. Specifically, the hemispheric differences in the functional interplay between regions whose asymmetries are altered by AD have yet to be examined. Furthermore, previous studies focused on structural asymmetries in patients with AD and ignored the relationship between pathological asymmetries and clinical symptoms (Derflinger et al., 2011; Kutova et al., 2018; Tetzloff et al., 2018).

In this study, we assessed the hemispheric differences in structures and functional networks between patients with AD and HCs. We used VBM, which was adapted for brain lateralization analysis, to investigate structural asymmetries. Seed-to-whole-brain resting-state functional connectivity (RSFC) was then adopted to map the functional networks associated with asymmetrically altered regions. Simultaneously, we explored the correlation between such asymmetries and clinical symptoms of AD.

## Materials and Methods

### Participants

Forty-eight patients with AD were recruited in the study. These patients were clinically diagnosed by a specialist in accordance with the following NINCDS-ADRDA (McKhann et al., 1984) criteria: (a) meeting criteria for possible or probable AD, (b) mini-mental state examination (MMSE) score <24, and (c) clinical dementia rating (CDR) score ranging from 0.5 to 2. The exclusion criteria for this study were substance use disorder, other neurological disorders, and a life-threatening somatic disease.

Thirty-two matched HCs were included in this study. The HCs fulfilled the following criteria: cognitively normal, no neurological or psychiatric disorders, no psychoactive medication use, an MMSE score of 28 or higher, and a CDR score of 0.

All the participants were right-handed, and they or their guardians provided a written informed consent. The study was performed in accordance with the latest revision of the Declaration of Helsinki, and the experimental procedures had full ethical approval from the local ethics committees of Anhui Medical University. The demographic and the clinical variables are shown in Table 1.

**TABLE 1:** Demographic and clinical characteristics of the Alzheimer’s disease patients and healthy controls.

	AD	HC	χ^2/^*t*/*Z*	*p*-value
Sex (male/female)	28/20	17/15	0.212^a^	0.65
Age (years)	66(9.9)^b^	65(9.2)^b^	0.49^b^	0.63
MMSE score	15.5(4.7)^b^	28.4(1.5)^b^	−17.72^b^	<0.001
Hooper score	6.66(5.59)^b^	18.67(2.61)^b^	–12.38	<0.001
ADL score	29(11)^c^	20(0)^c^	−6.85^c^	<0.001
CDR score	1(1)^c^	0(0)^c^	−7.27^c^	<0.001
GDS score	4.0(0.5)^c^	2(1)^c^	−6.78^c^	<0.001

*^a^Pearson chi-square test. ^b^Normally distributed data are described as the mean (standard deviation) and compared by two-sample t-tests. ^c^Non-normally distributed data are described as the median (interquartile range) and compared by Mann–Whitney U-tests. AD, Alzheimer’s disease; HC, healthy control; MMSE, mini-mental state examination; Hooper, hooper visual organization test; ADL, activities of daily living scale; CDR, clinical dementia rating scale; GDS, global deterioration scale.*

### Neuropsychological Assessment

All the participants underwent a clinical evaluation and a neuropsychological assessment. The following neuropsychological tests were administered to each participant for the purpose of establishing a clinical diagnosis, as described previously (Lezak et al., 2004; Woodward et al., 2017): (i) general cognitive functions were assessed using the MMSE (Burns, 1998), (ii) the CDR (Morris, 1993) and the Global Deterioration Scale (GDS) were used as a proxy of disease severity, (iii) daily function was assessed using the Lawton–Brody Activities of Daily Living (ADL) scale (Salmon and Bondi, 2009), and (iv) the Hooper Visual Organization Test (Hooper) was adopted to measure the participants’ ability to organize visual stimuli (Lopez et al., 2003). Testing was conducted by board-certified neuropsychologists and research staff.

### MRI Data Acquisition

All MRI data were acquired at The First Affiliated Hospital of Anhui Medical University. The participants were asked to relax and to keep their eyes closed, but to remain awake, and not to think of anything during the MRI acquisition (Wang et al., 2017). Structural MRI and functional MRI (fMRI) for each participant were performed using a 3-T scanner (Signa HDxt, GE Healthcare, Buckinghamshire, United Kingdom). Sagittal 3D high spatial resolution T1-weighted images were acquired using a brain volume sequence with the following parameters: repetition time/echo time ratio = 8.676/3.184 ms, inversion time = 800 ms, flip angle = 8, field of view = 256 mm × 256 mm, matrix size = 256 × 256, slice thickness = 1 mm, voxel size = 1 mm × 1 mm × 1 mm, and number of sections = 188. The resting state fMRI (rs-fMRI) images were acquired using a standard echo planar imaging sequence: repetition time/echo time ratio = 2,000/22.5 ms, 240 volumes, flip angle = 30°, 33 slices, thickness/gap = 4.0/0.6 mm, voxel size = 3.4 mm × 3.4 mm × 4.6 mm, matrix size = 64 × 64, and field of view = 220 mm × 220 mm.

### Calculation of Structural Asymmetries

Structural MRI data were pre-processed using the VBM8 toolbox^1^, a software package based on Statistical Parametric Mapping software (SPM 8,^2^). All T1-weighted images were affinely registered to standard space and segmented into gray matter (GM), white matter (WM), and cerebrospinal fluid. Next, the segmented images were spatially normalized using symmetric tissue probability maps (downloaded from^3^) by DARTEL algorithm (Liu et al., 2012, 2019). The original GM and WM images were flipped horizontally in the midsagittal plane [*x* = 0; i.e., left-right-flipped (LR-flipped)]; original and LR-flipped symmetric templates were created (Luders et al., 2004; Kurth et al., 2015). Using this symmetric template, we produced a right hemispheric (R Hip) mask for further data analysis. The asymmetry index (AI) of GM volume (GMV) asymmetries was determined using the formula: AI(r) = (R Hip r-maps minus LR-flipped L Hip r-maps). We then generated new AI maps. These AI maps were smoothened with a 4-mm full-width at half-maximum (FWHM) Gaussian kernel. In order to avoid the false transition of information (blurring) due to smoothing, we decided to keep the right hemisphere. A positive AI indicated a rightward asymmetry and a negative AI indicated a leftward asymmetry (Kurth et al., 2015). All preprocessing steps were based on the protocol generated by Kurth and colleagues (Kurth et al., 2015).

### rs-fMRI Data Preprocessing

Data Processing Assistant for Resting-State Functional MR Imaging toolkit, advanced edition, was used for rs-fMRI data processing. The first 10 time points were discarded to allow for magnetization equilibrium. The slice timing for the remaining images was corrected, and the images were realigned to the first volume to account for head motion. All the participants exhibited a maximum displacement of <3 mm and an angular motion of <3°. All fMRI images were normalized to the symmetric template described above and resampled at 3 mm × 3 mm × 3 mm (Yan et al., 2009; Kurth et al., 2015). Then, the functional images were linearly detrended, and the detrended images were smoothened using a Gaussian kernel of 4 mm FWHM. Subsequently, the functional images were filtered with a temporal band-path of 0.01–0.10 Hz, and six motion parameters, white matter, and cerebrospinal fluid signals were regressed out. Because previous studies have shown that global mean signal regression can lead to spurious resting-state functional correlations and false inferences, particularly at the group level, the global mean signal was not regressed during preprocessing in this study (Gotts et al., 2013; Saad et al., 2013; Wang et al., 2017).

### RSFC Analyses and AI of RSFC

The brain regions with significant structural asymmetry alterations were adopted as seed regions; then, a seed-based whole-brain functional connectivity analysis was performed. Pearson’s correlation analysis was carried out between the mean time series for each seed region and each voxel of the whole brain. Following the protocol described above, *r*-maps for each subject were generated.

Determining the AIs of RSFC analyses was more complex than for structural asymmetry. Unilateral structures displaying asymmetric alterations exhibit FC with brain regions of both the ipsilateral and the contralateral hemispheres (Yan et al., 2009). The AI of FC was defined as the difference between the FC of a seed region in the right hemisphere (RH) and other cerebral regions; the FC of the counterpart seed region in the left hemisphere (LH) was defined similarly (Yan et al., 2009). A positive value for an AI_(r)_ map represented a rightward asymmetry, which indicated that the connectivity of the RH seed region was greater than the connectivity of the LH seed region with its counterpart. By contrast, a negative value for an AI_(r)_ map represented a leftward asymmetry, which indicated that the connectivity of the LH seed region was greater than the connectivity of the RH seed region with its counterpart. An example is illustrated below, indicating how FC in hemispheric asymmetry was examined.

**FIGURE 1:**
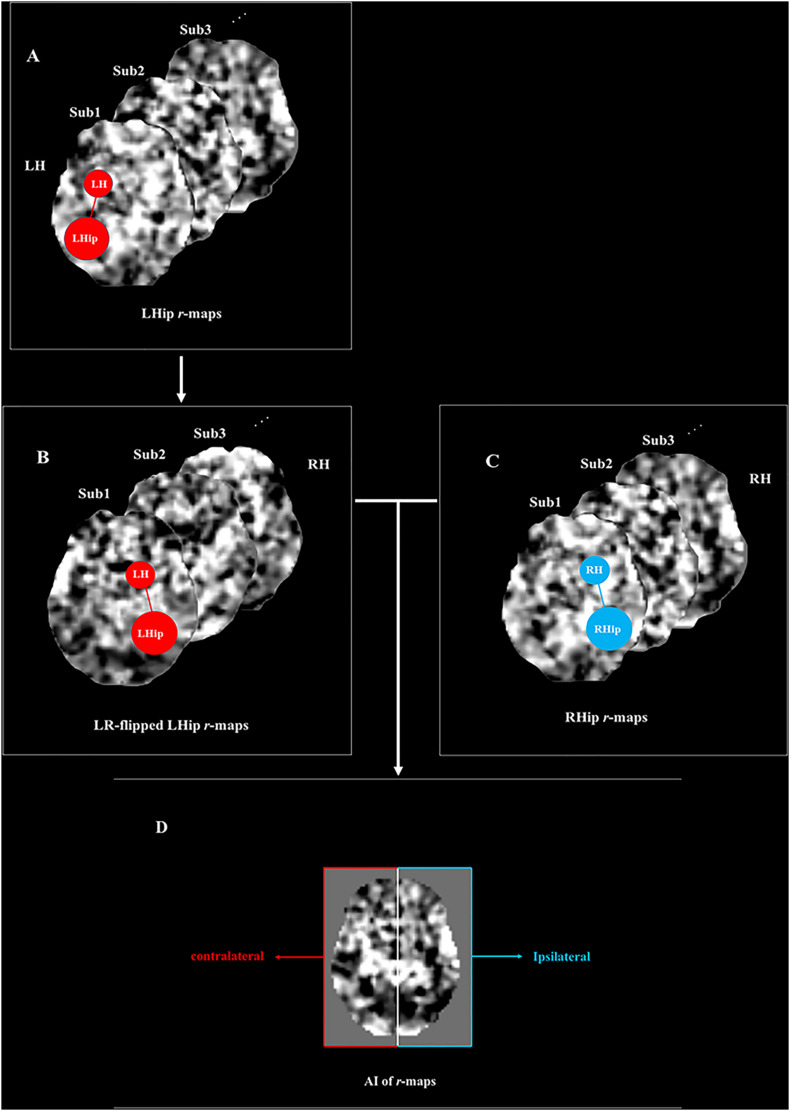
The diagram of asymmetry index (AI) calculates the function connectivity (FC) between the hippocampus and insula. **(A)** Individual left hippocampus r-maps (L-r maps). The schema on the first r-map represented the functional connectivity of the left hippocampus with the brain regions in the left hemisphere (i.e., LHip—LH). **(B)** LR-flipped left hippocampus r-maps (LR-r maps). As indicated by the schema, the connectivity of the left hippocampus with its ipsilateral brain regions was flipped to the right side and the connectivity of the left hippocampus with its contralateral brain regions was flipped to the left side. **(C)** Individual right hippocampus r-maps (R-r maps). The schema represents the functional connectivity of the right hippocampus with the brain regions in the right hemisphere (i.e., RHip—RH) **(D)** Individual AI(r) maps were calculated by the formulation. The formulation equaled (R-r maps - LR-r maps)/(R-r maps + LR-r maps). The right side of the AI(r) map represented the asymmetry of functional connectivity of the hippocampus with its ipsilateral hemisphere (i.e., RHip—RH vs LHip—LH), and the left side of the AI(r) map represented the asymmetry of functional connectivity of the amygdala with its contralateral hemisphere (i.e., RHip—LH vs LHip—RH). The positive value of the AI(r) map represented a rightward asymmetry which meant the connectivity of right hippocampus with such region was greater than the connectivity of left hippocampus with its counterpart. On the other hand, the negative value of the AI(r) map represented a left asymmetry, which meant that the connectivity of the left hippocampus with such region was greater than the connectivity of the right hippocampus with its counterpart. LHip, left hippocampus; RHip, right hippocampus; LH, left hemisphere; RH, right hemisphere. The figure was adapted from Yan et al. (2009).

Taking the RSFC between the hippocampus and the insula as an example, the right hippocampus (RHip) and the left hippocampus (LHip) have FC with both the right insula (R-insula) and the left insula (L-insula). To investigate the hemispheric asymmetry of the RSFC between the hippocampus and the insula, a comparison should be performed between the FC of the RHip-R-insula and that of the LHip-L-insula or between the FC of the RHip-L-insula and that of the LHip-R-insula (Yan et al., 2009). To implement these comparisons, the RHip and the LHip should be set as the seed region, after which LHip *r*-maps and RHip *r*-maps for each subject would be generated. A new set of *r*-maps would then be generated by left-right-flipping the individual LHip *r*-maps, namely, the LR-flipped LHip *r*-maps. The AI of the RSFC for each subject would then be calculated using the formula: AI(r) = (RHip r-maps minus LR-flipped LHip r-maps). The right side of the *r*-map would represent the asymmetry of the FC between the hippocampus and the ipsilateral hemisphere, and the left side would represent the asymmetry of the FC between the hippocampus and the contralateral hemisphere. A positive value of the AI_(r)_ map would represent a rightward asymmetry, which would indicate that the connectivity between the RHip and the R-insula was greater than the connectivity between the LHip and the L-insula. By contrast, a negative value of the AI_(r)_ map would represent a leftward asymmetry, which would indicate that the connectivity between the LHip and the L-insula was greater than the connectivity between the RHip and the R-insula (Figure 1).

The AI_(*r*)_ maps were subjected to one-sample *t*-tests in a voxel-wise manner to determine the brain regions that exhibit significant asymmetries in the AD and the HC groups. The brain regions with a cluster-level familywise error rate (FWE)-corrected threshold of *p* < 0.05 (uncorrected voxel-level *p* < 0.01) were utilized as a mask. The comparison of the asymmetric alterations of amygdala-based FC between AD patients and HCs was limited to the brain areas determined as significant by one-sample *t*-tests of AD or HC data.

### Statistical Analyses

Clinical and demographic data were analyzed using the Statistical Package for the Social Sciences 20.0 (SPSS, Chicago, IL, United States). Age, sex, years of education, and total intracranial volume were included as covariates in both the structural and the FC analyses. Independent two-sample *t*-tests were conducted for *post hoc* comparisons of the AI of the structural and the FC data. The signals of brain regions with significant differences were extracted for further analysis.

For the MRI analysis, to explore differences in the AI of structures and FC between groups, a permutation test was performed for multiple comparisons using the Statistical non-Parametric Mapping program 13^4^, a toolbox for use with the software SPM 12 (Gutierrez-Barragan et al., 2017). The cluster-level FWE was used in multiple comparisons to reduce type I error (threshold, *p* < 0.05; cluster-forming threshold at voxel level, *p* < 0.01).

We performed correlation analyses and multiple linear regression between AI values based on structure and FC and the participants’ clinical data to further explore whether neuroimaging indices were related to symptom severity. The significance level was set at *p* < 0.05.

## Results

### Demographic and Clinical Characteristics

There were no significant differences in age or sex between the AD and the HC groups. As expected, scores on the MMSE, CDR, GDS, ADL, and Hooper scales were markedly different between the two groups, with significantly worse performance in the AD group compared to the HCs. The demographic and the clinical characteristics of the study participants are summarized in Table 1.

### Structural Asymmetries

**FIGURE 2:**
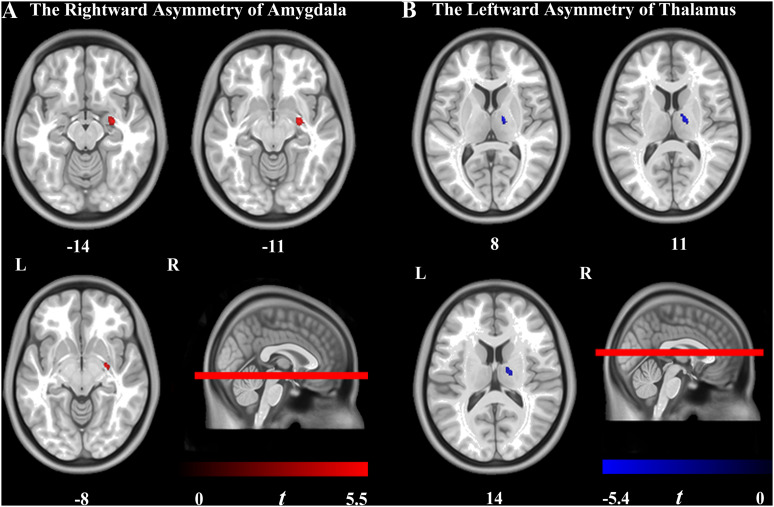
Brain regions showing the significant hemispheric asymmetry of VBM. **(A)** Rightward lateralization was found in the amygdala in the Alzheimer’s disease (AD) group. **(B)** Leftward lateralization was found in the thalamus in the AD group. Cluster-level familywise error rate-corrected *p* < 0.05 (uncorrected voxel-level *p* < 0.001; cluster size > 30 voxels) was utilized. The color bar indicates the t-value. L, left hemisphere; R, right hemisphere.

**FIGURE 3:**
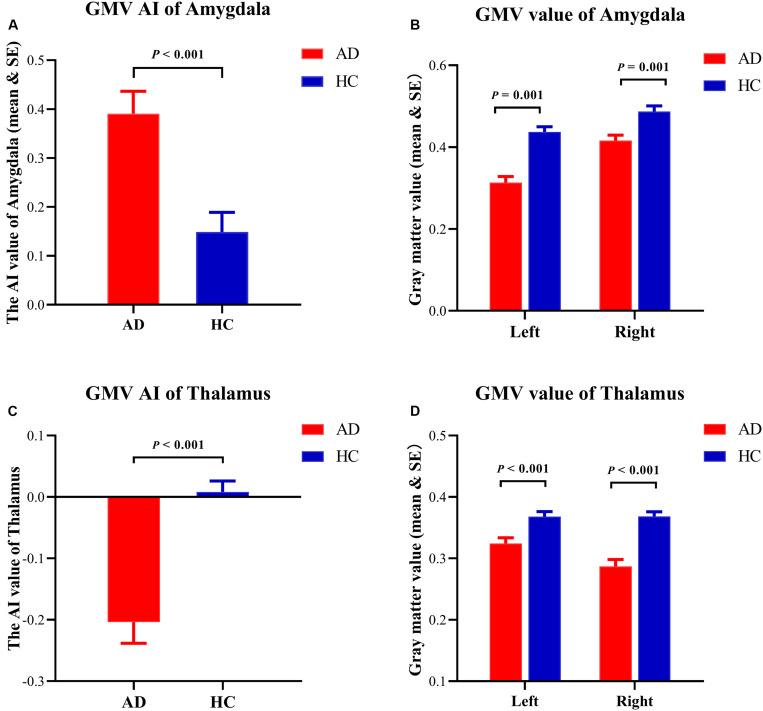
Details of structural asymmetries. **(A)** The asymmetry index (AI) value of the amygdala was greater in the Alzheimer’s disease (AD) group, which represented a rightward lateralization in AD. **(B)** The gray matter volume (GMV) of the amygdala showing the atrophy in both hemispheres in the AD group. **(C)** The AI value of the thalamus was reversed in the AD group, which represented a leftward lateralization in the AD group. **(D)** The GMV of the thalamus showing the atrophy in both hemispheres in the AD group.

The results showed that the rightward asymmetry of the amygdala (130 voxels, peak location: *x* = 27, *y* = −8, *z* = −12, peak intensity = 5.45, and cluster-level FEW-corrected *p*-value = 0.031) and the leftward asymmetry of the thalamus (162 voxels, peak location: *x* = 15, *y* = −9, *z* = 9, peak intensity = 5.43, and cluster-level FEW-corrected *p*-value = 0.033) were significantly higher in the AD group compared to the HCs. Furthermore, the GMV of the bilateral amygdala and the thalamus was significantly smaller in the AD group than in the HCs, but atrophy of the left amygdala and the right thalamus was more severe than that in contralateral regions in the AD group (Figures 2, 3).

### Asymmetric Alterations of FC

**FIGURE 4:**
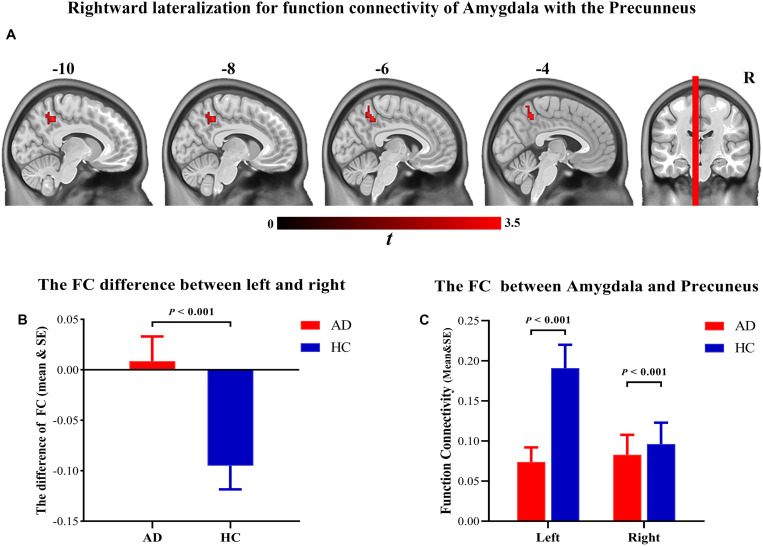
**(A)** Brain regions showing the significant hemispheric asymmetry of functional connectivity with amygdala. **(B)** Rightward lateralization for function connectivity of amygdala with the precuneus. **(C)** The function connectivity of the amygdala with the precuneus was significantly decreased in the Alzheimer’s disease patients.

Significant differences were observed between patients with AD and HCs in the FC between the amygdala and the precuneus (50 voxels, peak location: *x* = −6, *y* = −48, *z* = 45, cluster-level FEW-corrected *p*-value = 0.005). The asymmetry was reversed in patients with AD, which may be due to the significant decrease in FC between the left amygdala and the precuneus (Figure 4).

### Correlation and Multiple Linear Regression Analyses

**FIGURE 5:**
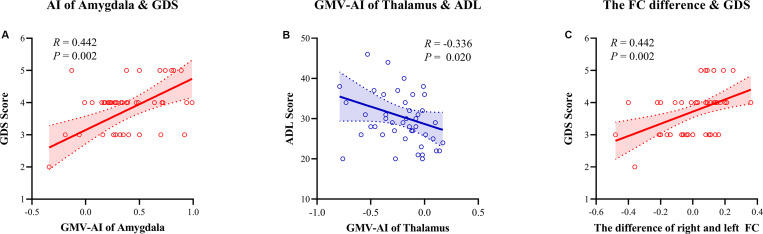
Spearman correlation analysis between the asymmetry index (AI) of gray matter volume (GMV)/function connectivity and neuropsychology assessment. **(A)** The AI of the amygdala GMV was positively correlated with the Global Deterioration Scale (*r* = 0.442, *p* = 0.002). **(B)** The AI of the thalamus GMV was negatively correlated with the Activities of Daily Living scale (*r* = –0.336, *p* = 0.020). **(C)** The AI of connectivity of the amygdala with the precuneus was positively correlated with the GDS scale (*r* = 0.442, *p* = 0.002). AI, asymmetry index; GMV, gray matter volume; FC, function connectivity; GDS, Global Deterioration Scale; ADL, Activities of Daily Living.

The correlation analyses revealed that the GMV AI values of the amygdala were positively correlated with the GDS scores (*r* = 0.442, *p* = 0.002), and the GMV AI values of the thalamus were negatively correlated with the ADL scores (*r* = −0.336, *p* = 0.020) in patients with AD. The AI values of the FC between the amygdala and the precuneus were significantly correlated with the GDS scores (*r* = 0.442, *p* = 0.002). No similar trend was observed in the HC group. In other words, a rightward asymmetry indicated greater disease severity. The multivariate linear regression demonstrated that the GDS scores were predicted (*R*^2^ = 0.232, *F*(1,47) = 14.187, *p* < 0.001; Figure 5) by the AI of the amygdala (β = 1.608; *t* = 3.767, *p* = 0.01), but not by the AI of the thalamus or FC (*p* > 0.05).

## Discussion

Changes in the asymmetric structure and the function of the human brain may be indicative of neurological disorders. We examined the degree of whole-brain asymmetry at the voxel level in patients with AD. First, we showed that the bilateral amygdala of the patients with AD exhibited asymmetric atrophy with rightward lateralization. Second, we revealed a leftward lateralization of thalamic atrophy. Furthermore, we observed a rightward asymmetry in FC between the amygdala and the ipsilateral precuneus, which is the opposite of what was observed in the HCs. Moreover, these lateral changes were associated with the clinical manifestations of AD. This suggests that lateralization of structure and function could be a useful biomarker for AD.

The rightward lateralization of the amygdala in patients with AD may result from severe contralateral atrophy of the amygdala as this was positively correlated with the severity of AD. In this study, we demonstrated that the GMV of the left amygdala was smaller than that of the right amygdala in the AD group. These results are in agreement with previous studies that have shown a faster progression of left hemispheric atrophy in patients with AD (Thompson et al., 2003, 2007; Damoiseaux et al., 2009; Donix et al., 2013; Li et al., 2016; Wessa et al., 2016; Sarica et al., 2018). Our study adds to previous work that has revealed hemispheric asymmetries in the amygdala of patients with AD (Muller et al., 2005; Shi et al., 2009; Minkova et al., 2017; Sarica et al., 2018). Although the degree of lateralization of the amygdala increased in patients with AD, the volumes of both the left and the right amygdala were atrophied compared with those of HCs, consistent with previous studies that indicated global cerebral atrophy associated with disease progression (Ohnishi et al., 2001; Remy et al., 2005; Nho et al., 2012; Matsuda, 2013, 2016). Both the correlation and the multivariate linear regression analyses demonstrated that the AI values of the amygdala were positively correlated with disease severity. Accordingly, we hypothesized that, due to the rapid atrophy of the left amygdala, its structural asymmetries became more pronounced, and these changes were associated with worse clinical symptoms in patients with AD.

The lateralization of the thalamus of patients with AD was also reversed compared with those of HCs, and this was related to the degree of functional impairment. Patients with AD displayed a significantly smaller thalamus on the right side, contrary to previous reports (Low et al., 2019). Low et al. (2019) revealed no significant difference in the size of the whole thalamus but observed left lateralization in the ventral thalamus of patients with AD. This discrepancy may be explained by the fact that the previous study’s analysis was based on data collected at the ROI level, targeting the subnuclei of the thalamus. By contrast, a fully automated voxel-by-voxel analysis technique was adopted in the current study, which enabled an examination of cerebral asymmetries across the entire brain while avoiding the subjectivity of ROI-based approaches (Luders et al., 2004). It is worth noting that the leftward lateralization of the thalamus was more conducive to functional retention in patients with AD, consistent with a previous study (Bruen et al., 2008). This may be due to thalamic involvement in complex sensorimotor functions (Schmahmann, 2003).

The FC between the amygdala and the whole brain dramatically changes in patients with AD. Compared with HCs, lateralization of FC of the amygdala with the precuneus was reversed in the AD group, which has never been reported previously in the literature. The results imply that the rightward dominance in FC in the AD group was primarily due to a significant decrease in FC between the left amygdala and the precuneus. These results agree with positron emission tomography studies showing asymmetric metabolism between hemispheres (Loewenstein et al., 1989; Weise et al., 2018). Previous studies have shown abnormal activation and FC between the whole brain and the precuneus in patients with AD (Zhang et al., 2010; Anticevic et al., 2012; Mistridis et al., 2014; Raichle, 2015). However, the abnormal interactions between the amygdala and the precuneus observed in our study have not been reported in previous works, possibly because most studies based their analyses on specific isolated ROIs (Minkova et al., 2017; Sarica et al., 2018).

The abnormalities in the asymmetry of FC between the amygdala and the precuneus were associated with disease severity in patients with AD. The AI values for the FC between the amygdala and the precuneus were positively correlated with the scores on the GDS. In general, these data indicate that the greater the degree of rightward lateralization of the function of the amygdala, the more serious the severity of AD is. This may be because the amygdala is involved in multiple social and cognitive functions, including learning and memory (Janak and Tye, 2015; Rutishauser et al., 2015; Haller, 2018), and dysfunctions of the amygdala might lead to functional degeneration. Furthermore, the present findings support the notion that distinct asymmetries of brain atrophy in AD may represent etiologically distinct phenotypic subgroups (Giannakopoulos et al., 1994; van Dyck et al., 1998).

Unlike a previous study, we did not find asymmetric alterations in hippocampal volume. There are many possible reasons for this. One possibility is that the participants of the current study were at the moderate–severe stages of AD. Hippocampal atrophy has been observed in the preclinical stage of AD, and a progressive decrease in the degree of asymmetry has been reported between mild cognitive impairment and AD (Liu et al., 2018). In the moderate–severe stages of AD, the bilateral hippocampi become severely atrophied, which may be why asymmetric differences in the hippocampus have not been observed in some cases (Kim et al., 2012); the findings by La Joie et al. (2013) also support this possibility. Similarly, differences between the early and the moderate–severe stages of the disease can also be observed in the thalamus (Low et al., 2019). Different analysis methods may also underlie the observed discrepancies as previous studies are based on ROIs, which is typically a more subjective approach (Luders et al., 2004). Moreover, differences in the templates used may be another reason as the amygdala is anatomically adjacent to the hippocampus. In our study, different results were seen, depending on the brain atlas used to identify the structures. A smaller cluster was observed in the hippocampus when the Automated Anatomical Labeling atlas was used to identify the structures, but this was not seen when using the Harvard–Oxford brain atlas, and the cluster was too small (17 voxels) to exclude the possibility of this representing noise rather than a true signal. Thus, we did not report this cluster in the results. In the future, more accurate segmentation methods, more accurate brain templates, and longitudinal studies will be needed to validate these findings.

Although this study presents several meaningful findings, there were several limitations to consider. First, this was a cross-sectional study. Second, the number of patients with AD was small. Larger sample sizes are needed to further validate the generalizability of the findings of this study. Third, the study did not conduct a stratified analysis based on the severity of the disease. Further research should be conducted to investigate whether the measure of morphometric asymmetry could potentially be used to monitor disease progression and predict clinical outcomes.

## Conclusion

Overall, our study highlights the value of considering cerebral asymmetries in clinical evaluations and their relevance to clinical measures, such as GDS and ADL assessments. The results indicated that the etiology of AD is not associated with isolated abnormal activity in individual brain structures or regions but that the alterations are highly functionally related between cerebral structures/regions. These results provide insight into the neural mechanisms of AD and the asymmetries that develop alongside the pathophysiology of disease progression, not only in terms of structural asymmetries but also in terms of asymmetries between functional networks, which may be useful as potential biomarkers of AD.

## Data Availability Statement

The datasets generated for this study are available on request to the corresponding author.

## Ethics Statement

The studies involving human participants were reviewed and approved by the Ethics Committees of the Anhui Medical University. The patients/participants provided their written informed consent to participate in this study.

## Author Contributions

XW and YW performed the analysis and wrote the manuscript. ZG, SZ, and LW helped to collect behavioral and imaging the data. G-JJ helped in the MRI data analysis. YT and KW designed and supervised the study. All authors contributed to the article and approved the submitted version.

## Conflict of Interest

The authors declare that the research was conducted in the absence of any commercial or financial relationships that could be construed as a potential conflict of interest.

## Footnotes

1 http://dbm.neuro.unijen-a.de/vbm/download/

2 http://www.fil.ion.ucl.ac.uk/spm

3 http://dbm.neuro.unijena.de/vbm8/TPMsymmetric.nii

4 http://www2.warwick.ac.uk/fac/sci/statistics/staff/academicresearch/nichols/software/snpm
