# Supplementary material for: Asymmetric Differences in the Gray Matter Volume and Functional Connections of the Amygdala Are Associated With Clinical Manifestations of Alzheimer’s Disease
Source: Front Neurosci. 2020 Jun 26;14:602. doi: 10.3389/fnins.2020.00602 (PMC7332559; doi:10.3389/fnins.2020.00602)
Supplement: Supplementary file 1 [file Presentation_1.pdf]

Supplementary material

The results of non-parametric permutation test for one-sample test

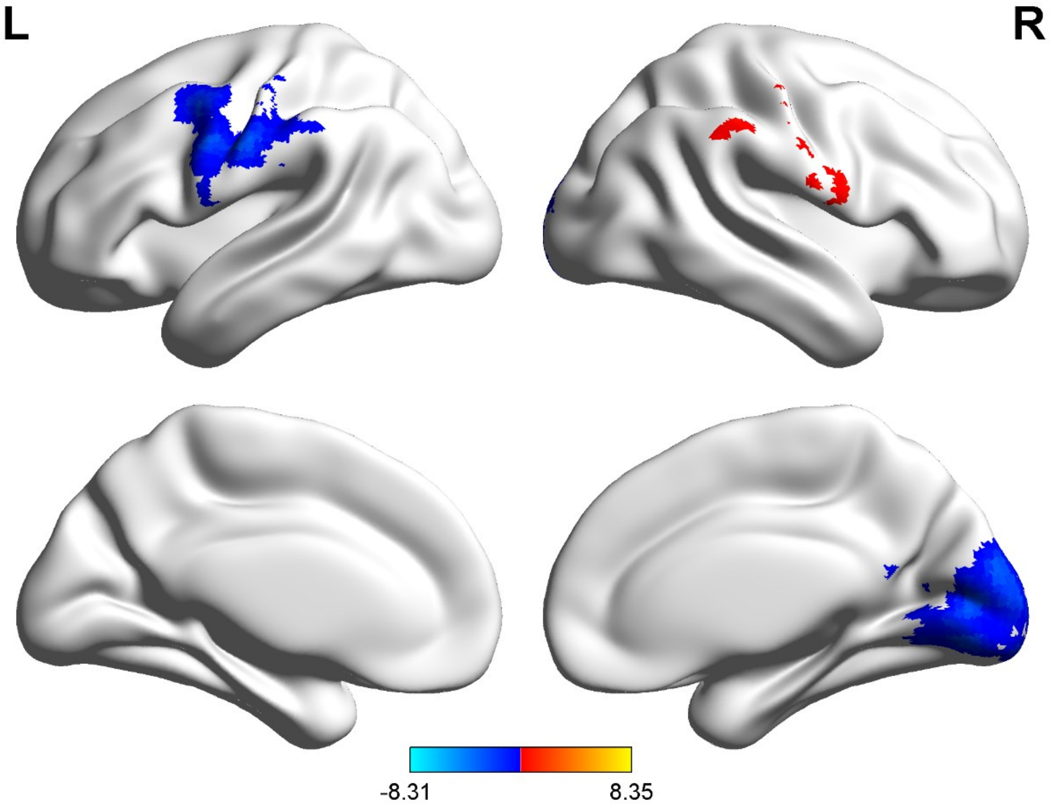

Figure S1

Figure S1: Brain regions showing significant asymmetries for FC of amygdala in AD group. The FC of ipsilateral occipital lobe with amygdala and the FC of contralateral temporal and frontal lobe with amygdala showed leftward asymmetries. The FC of ipsilateral temporal lobe with amygdala showed rightward asymmetries

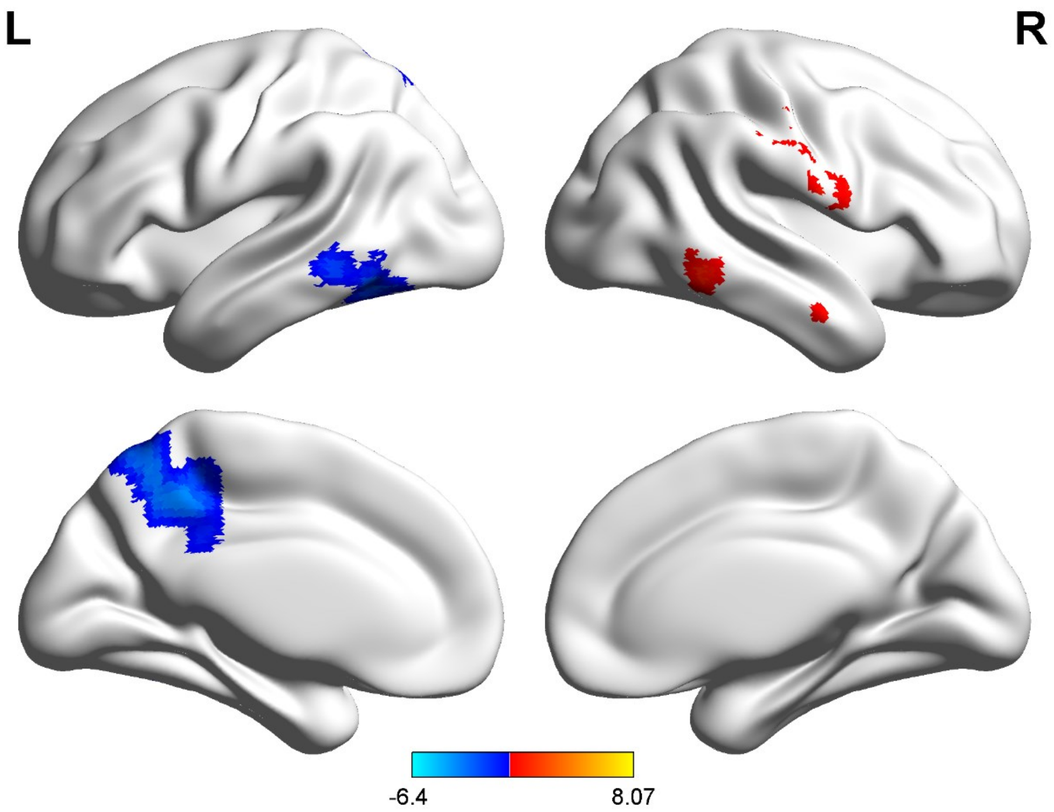

Figure S2

Figure S2: Brain regions showing significant asymmetries for FC of amygdala in HC group. The FC of contralateral precuneus with amygdala and the FC of contralateral temporal lobe with amygdala showed leftward asymmetries. The FC of contralateral temporal lobe with amygdala showed rightward asymmetries

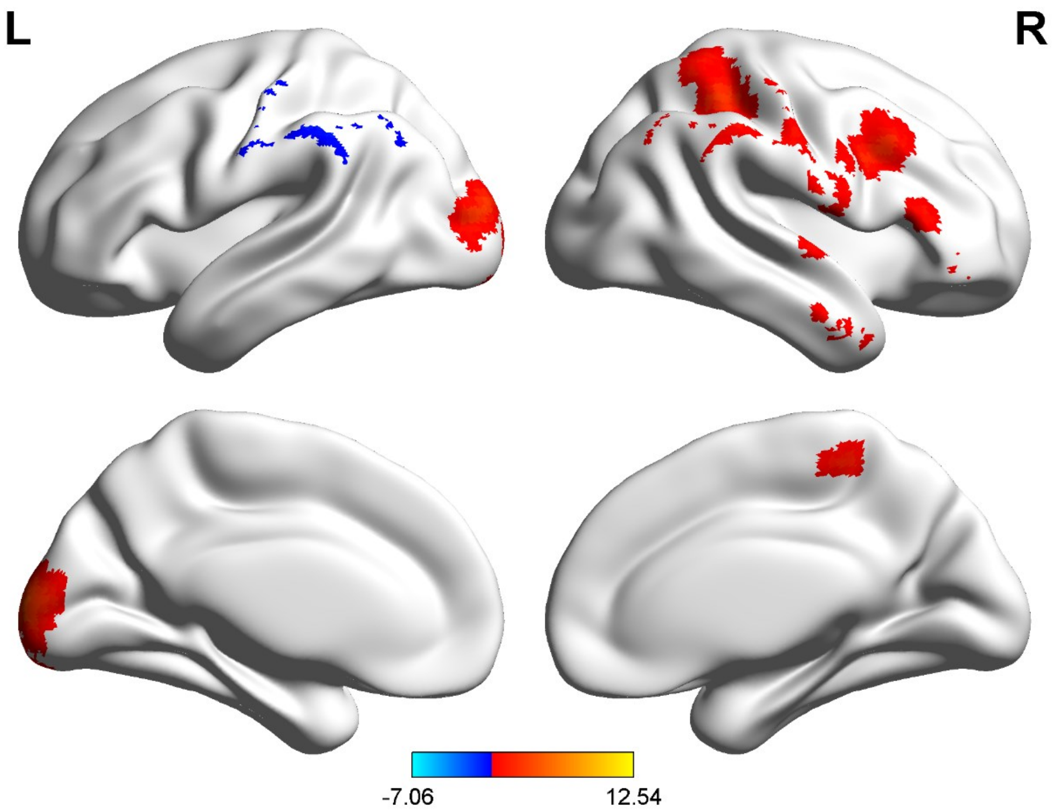

Figure S3

Figure S3: Brain regions showing significant asymmetries for FC of thalamus in AD group. The FC of contralateral temporal lobe with thalamus showed leftward asymmetries. The FC of contralateral occipital lobe with thalamus and the FC of ipsilateral precuneus, frontal, temporal and parietal lobe with thalamus showed rightward asymmetries.

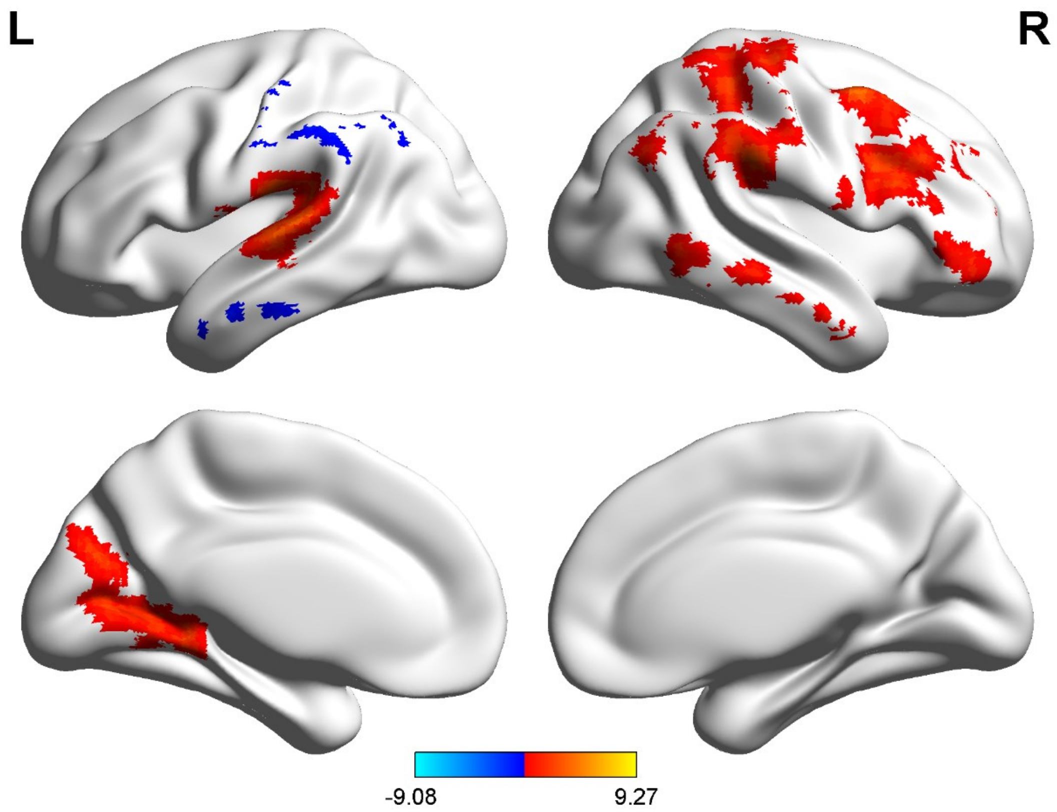

Figure S4

Figure S4: Brain regions showing significant asymmetries for FC of thalamus in HC group. The FC of contralateral temporal lobe with thalamus showed leftward asymmetries. The FC of contralateral occipital and temporal lobe with thalamus and the FC of ipsilateral frontal, temporal and parietal lobe with thalamus showed rightward asymmetries.
